# Role and Regulation of Cytokinins in Plant Response to Drought Stress

**DOI:** 10.3390/plants9040422

**Published:** 2020-03-31

**Authors:** Nguyen Ngoc Hai, Nguyen Nguyen Chuong, Nguyen Huu Cam Tu, Anna Kisiala, Xuan Lan Thi Hoang, Nguyen Phuong Thao

**Affiliations:** 1 Applied Biotechnology for Crop Development Research Unit, School of Biotechnology, International University, Ho Chi Minh City 700000, Vietnam; hainn.ssc@gmail.com (N.N.H.); nguyenchuong1402@gmail.com (N.N.C.); mikanguyen1201@gmail.com (N.H.C.T.); 2 Vietnam National University, Ho Chi Minh City 700000, Vietnam; 3 Environmental and Life Science, Trent University, Peterborough, ON K9L 0G2 Canada; 4 Department of Biology, Trent University, Peterborough, ON K9L 0G2, Canada; annakisiala@trentu.ca

**Keywords:** abscisic acid, CK metabolic genes, CK signaling genes, cytokinin, drought tolerance

## Abstract

Cytokinins (CKs) are key phytohormones that not only regulate plant growth and development but also mediate plant tolerance to drought stress. Recent advances in genome-wide association studies coupled with *in planta* characterization have opened new avenues to investigate the drought-responsive expression of CK metabolic and signaling genes, as well as their functions in plant adaptation to drought. Under water deficit, CK signaling has evolved as an inter-cellular communication network which is essential to crosstalk with other types of phytohormones and their regulating pathways in mediating plant stress response. In this review, we revise the current understanding of CK involvement in drought stress tolerance. Particularly, a genetic framework for CK signaling and CK crosstalk with abscisic acid (ABA) in the precise monitoring of drought responses is proposed. In addition, the potential of endogenous CK alteration in crops towards developing drought-tolerant crops is also discussed.

## 1. Introduction

### 1.1. Drought Stress—The Most Common Abiotic Threat to Plant Performance 

During their life cycle, plants are constantly exposed to a changing environment, often including various adverse biotic and/or abiotic factors [1,2,3]. Such stress conditions may prevent plants from fulfilling their maximum potential performance, even threatening their survival. Environmental disturbance caused by human activities, especially in recent decades, has exacerbated the frequency of abiotic stresses, thus increasing the difficulties in securing adequate food supply [4]. Amongst these, drought and salinity are often considered as the most serious factors limiting crop production [5]. Particularly, drought adversely affects numerous biological activities, such as photosynthesis, nutrient acquisition and cellular metabolism [6], as a result of the decrease in chlorophyll concentration, cell membrane stability, leaf water content and the increase in oxidation-induced damage [7,8]. It has been known that plants respond to drought in order to alleviate the stress effects on their growth and productivity [9]. These responsive activities are related to morphological, physiological, biochemical and molecular changes in plants, which are outcomes of the capacity of the plant to perceive and transduce the stress signal to regulate their gene expression [6,10,11,12]. 

### 1.2. A Portrait of CKs in Plant Development and Stress Adaptation

**Figure 1:**
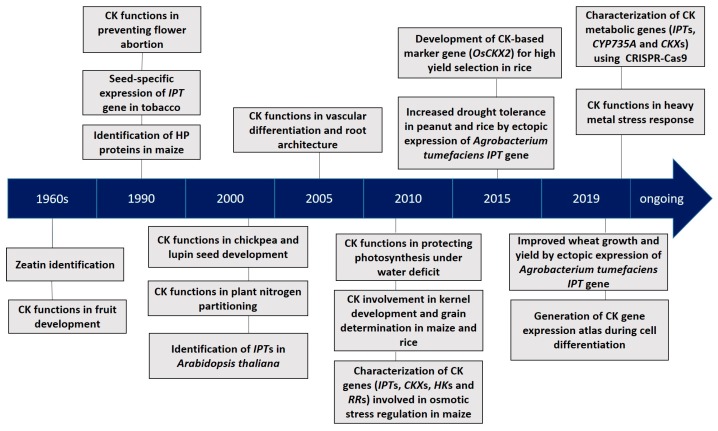
A timeline of key progresses in cytokinin (CK) research [23,24,25,26,27,28,29,30,31,32,33,34,35,36,37,38,39,40,41,42,43,44,45]. HP: Histidine phosphotransfer; IPT: isopentenyl transferase.

Cytokinins (CKs) are a class of the multifaceted plant hormone that plays essential roles in a wide array of processes of plant growth and development [13]. Since the discovery of their existence in maize (*Zea mays*) seeds over 50 years ago, CKs have been extensively studied for their chemical nature, metabolism and signal transduction pathway, as well as their functions in plant growth and development (Figure 1). Significant regulatory functions of CKs at the tissue and organ levels include the inhibition of lateral root initiation [14], differentiation of phloem and metaxylem in roots [15], regulation of cell division, photomorphogenic cell differentiation in expanding leaves and shoots [16,17], and inhibition of leaf senescence [18]. 

Endogenous CKs are adenine derivatives with isoprenoid or aromatic side chains. Depending on hydroxylation or reduction of the side chain, isoprenoid CKs, which are widespread in nature, can be distinguished as isopentenyladenine (iP)-, *trans*-zeatin (*t*Z)-, *cis-*zeatin (*c*Z)- or dihydrozeatin (DHZ)-type derivatives. By contrast, aromatic CKs, such as N6-(meta-hydroxybenzyl)adenine (BA), are found in plants at a lower abundance [19]. The isoprenoid CKs differ in their biological functions, biochemical properties, metabolic conversions, and their transportability across the plant body [13]. Various enzymes involved in CK metabolism, including CK biosynthesis, inter-conversion between CK types and CK degradation, are responsible for maintaining CK homeostasis [20]. CK metabolism has been extensively explored to understand the function of a large number of genes and enzymes, as well as the composed metabolic network regulated by CKs throughout the plant kingdom (reviewed by Pavlů et al. [13]; Zwack et al. [18]). CK signaling pathway is also triggered by various environmental stimuli, such as changes in temperature, nutrition levels and osmotic conditions, whereby the phospho-relay cascades of the two-component system (TCS) (i.e., downstream components of CK signaling, which will be discussed in detail in Section 2.2), are initiated and lead to the expression regulation of specific genes involved in plant adaptation [13,20]. More recently, CK crosstalk with ethylene (ET), jasmonates (JAs), salicylic acid (SA) and abscisic acid (ABA) have been recognized [17,20,21,22], indicating a coordinating network among these phytohormones in plant stress tolerance. Furthermore, CK biosynthesis and signaling components are known to act as constitutive signals defining plant response to drought stress and regulating plant drought acclimation [13]. Their rapid responses, spatiotemporal expression, as well as their widely associated pathways, make CKs excellent candidates to regulate complex morphogenetic processes under water deficit. Therefore, in this review, we will emphasize and propose a model for the role and regulation of plant CKs under drought stress.

## 2. CK Regulation in Plant Response to Drought

CKs naturally occur in over 25 forms [46], which are distributed and translocated differently among plant species and development stages. Recent studies suggest that the bioactive function and translocation of CKs are mainly attributed to free-base forms (e.g., iP, *tZ*), rather than other forms of ribosides and ribotides [47,48,49,50]. Studies of substrate specificity and sensitivity of CK receptors clearly confirm that the free-base forms display distinct preferences for binding toward different CK receptors and have the highest affinity to histidine kinase (HK) domain [51,52,53]. Adding to the detailed knowledge of biosynthesis, signal perception and transduction, transport and hormonal crosstalk of CKs, many excellent publications have described the functions of CKs in plant development [45,54,55]. CK response and translocation are not only controlled by cellular signaling, but also associated with stress stimuli, to shape molecular and biochemical responses in ways that promote the adaptation to environmental changes, including source/sink modification [56], delayed senescence [57], and grain yield [58,59]. Supporting evidence has been accumulating to show possible CK crosstalk with other phytohormones, including auxin, SA and brassinosteroids (BR) [60,61], and with ABA [62] in particular, in mediating drought stress response. 

### 2.1. Response of CK Metabolic Genes to Drought

In plants, CK metabolism is generally regulated by adenosine phosphate-isopentenyl transferases (IPTs) and CK oxidases/dehydrogenases (CKXs). The former are encoded by *IPT* genes and responsible for CK biosynthesis. There are two groups of IPTs affecting CK biosynthesis in *Arabidopsis thaliana*, including seven *ATP/ADP IPT* gene family members (GFMs) (*IPT1*, *IPT3*, *IPT4*, *IPT5*, *IPT6*, *IPT7* and *IPT8*) and two *transfer RNA IPT* GFMs (*IPT2* and *IPT9*) [63]. Interestingly, nine *IPT* GFMs in *Arabidopsis* encode eleven IPT proteins. It has been found that *AtIPT9* has three alternative splicings, suggesting that *AtIPT9* could be a remarkable candidate for further studies on CK homeostasis [64]. Among those GFMs, *AtIPT3* and *AtPT9* expression was down regulated under drought conditions [64]. Since the application of whole-genome sequencing to a wider range of plant species, the *IPT* family has been successfully identified and annotated, with 9 members in maize [35], 14 members in soybean (*Glycine max*) [65], 10 members in rice (*Oryza sativa*) [66], and 12 members in apple (*Malus domestica*) [67]. An increasing number of investigations focus on the participation of *IPT* genes in the regulation of plant defense response to water deficit in crop species. For instance, *IPT* transcript abundance was reported to be associated with dehydration and drought stress conditions, during both vegetative and reproductive stages in soybean [65], cabbage (*Brassica rapa*) [68] and rice [64]. Unlike in *Arabidopsis*, a total of 10 *IPT* GFMs in rice encode 10 IPT proteins with no alternatively spliced isoforms. It has also been reported that dehydration induces the expression of *OsIPT5*, yet down-regulates the transcriptional activities of *OsIPT2* [64,69]. In soybean, the *GmIPT08* transcriptional expression was consistently increased in shoots and trifoliate leaves upon drought and dehydration treatments [65]. During the reproductive phase, *GmIPT08* and *GmIPT10* were found to positively respond to drought conditions [65]. Another study found the majority of *BrIPT* GFMs in Chinese cabbage to be initially up-regulated, before falling to basal levels during the extention of drought exposure, except for the *BrIPT7-1* gene, in which transcripts were continuously increased and maintained at high levels under severe drought conditions [68]. Such findings regarding expression data and functional characterization of *IPT* GFMs give support to the hypothesis that *IPT* genes possess a wide range of unique functions during drought stress (Table 1), which will be described in detail in Section 3. Therefore, a number of promising IPT candidates (such as *OsIPT05*, *GmIPT08*, *GmIPT10* and *BrIPT7-1*) for modulation by genetic engineering could be revealed to develop novel crop cultivars with higher drought tolerance. Interestingly, recent work reported that *Arabidopsis AtIPT3* gene is also induced by photosynthetically generated sugars under elevated carbon dioxide (CO_2_) in roots [70], thereby validating that *IPT*s should be further analyzed to deal with drought tolerance and productivity in the new era of climate change and the relentless rise of CO_2_ emmisions.

The other important component of CK metabolism is the CKX enzyme, which accounts for the irreversible inactivation of CKs by cleaving the side chain from CK molecules [46]. Genome-wide association studies have recently shed more light on the potential function of CKX members in plant species, with 13 *CKX* GFMs in maize [71], 11 *CKX* GFMs in rice [66], 12 *CKX* GFMs in apple [67], 11 *CKX* GFMs in wheat (*Triticum aestivum*) [72] and 23 *CKX* GFMs in rapeseed (*Brassica napus*) [73]. Increasing lines of evidence suggest that *CKX* GFMs play important roles in various plant physiological and developmental modifications under drought stress [74,75,76] (Table 1). Certain *CKX* GFMs also displayed significantly altered expression in response to drought and can be used as targets for genetic manipulation. For example, in soybean, *GmCKX07* and *GmCKX13* clearly showed higher transcriptional activities in roots and root hairs under limited water availability, suggesting that these GFMs are unique genetic resources that should be further studied to understand their involvement in plant drought adaptation [65]. A genome-wide association study in cabbage also found that three *CKX* GFMs, *BrCKX1-1*, *BrCKX1-2* and *BrCKX5*, maintained their higher transcription activities throughout drought treatment [68]. Furthermore, a study on the expression of 11 *CKX* members (from *SiCKX1* to *SiCKX11*) in germinating embryos of foxtail millet (*Setaria italica*) revealed that, except for *SiCKX2* and *SiCKX11*, the expression of *CKX* GFMs was up-regulated upon polyethylen glycol (PEG)-induced drought stress treatment and exogenous BA application [77]. These findings indicate that CKs play an important role in drought tolerance in foxtail millet via regulating CK dehydrogenase activities. To understand the regulatory role of CK dehydrogenase GFMs in woodland strawberry (*Fragaria vesca*), Jiang et al. (2016) identified a total of eight *FvCKX* genes from its genome [78]. Under drought stress, *FvCKX06* and *FvCKX8* positively responded in both shoots and roots, while *FvCKX1* and *FvCKX2* positively responded only in shoots. In addition, all of these GFMs also transcriptionally responded to ABA treatment [78]. As drought responses of CK metabolic genes vary by tissue types and the exposure time, future functional studies should provide a full inventory of CK metabolic genes, in relation to tissue-specific and spatially inducible expression, to reveal any additional roles of these genes in plant drought tolerance. In fact, there have been several studies demonstrating improved yield under drought conditions by modulating CK homeostasis, either via CK biosynthetic *IPT* GFMs [38,79] or CK degradation *CKX* GFMs [80]. 

### 2.2. Response of CK Signaling Genes to Drought Stress

CK signal transduction and response in plants are regarded as TCSs, which incorporate three signaling elements: receptor HKs, histidine phosphotransfer proteins (HPs) and CK response regulators (RRs). In this pathway, using *Arabidopsis* as the model, HPs (AHPs) are employed as direct downstream components of AHK receptors, to directly mediate phosphotransfer, which ramps up the function of type-B RRs (type B-ARRs) [81,82]. In this feedforward, type-B ARRs receive phosphoryl group from AHPs, that enable them to activate expression of downstream type-A *ARR* genes [83]. Additionally, the direct binding action of type-B ARRs to their target sites has been demonstrated *in planta* upon the application of CKs [84]. Consistent with having a crucial role in CK response [85] and plant development [86], type-B ARRs are associated with abiotic stress response, whereby they play a negative role in plant response to drought [87]. Unlike type-B ARRs, type-A ARRs repress the activity of type-B ARRs and act as a negative regulator in CK signaling [88]. These TCS components in the CK signaling are now gaining much attention in plant research, due to their contribution to multiple abiotic stress responses, through the control of hormonal cross-regulation mechanisms [89,90], lateral root initiation [91] and antioxidant defense [92]. Such attempts will facilitate the discovery of new TCS components involved in plant growth and development, as well as investigations on their possible contribution in drought stress response.

A number of transmembrane HKs are CK receptors, including AHK2, AHK3, and AHK4/CRE1 in *Arabidopsis*, are located mainly in the endoplasmic reticulum [93,94,95]. Further studies on the expression of *AHK2*, *AHK3* and *CRE1* revealed that they were significantly up-regulated upon exposure to dehydration conditions [96]. In maize, *ZmHK1* and *ZmHK3a*, two *HK* genes that are closely related to the *Arabidopsis CRE1* and *AHK2* respectively, were also found to be up-regulated by drought [97]. In soybean, gene expression analysis of 21 *GmHK*s revealed that *GmHK07*, *GmHK10* and *GmHK12* were significantly induced by dehydration conditions, suggesting the necessity for detailed functional studies of these genes to discover their role in mediating plant stress response [98]. In part of this study, transcripts of two *GmHP* genes (*GmHP03* and *GmHP06*) displayed higher levels under dehydration treatment [98]. Consistent with these reports, Thu et al. (2015) found that *GmHK07* was potentially associated with an enhanced drought tolerance phenotype after performing comparison in expression profiles of dehydration-responsive *TCS* genes between two Vietnamese soybean cultivars with contrasting drought-tolerant phenotypes [99]. Although more experimental evidence, such as *in planta* studies, is needed to support mechanistic models of GmHK07, it seems reasonable to assume that GmHK07 might be a key component in mediating drought tolerance in soybean.

CK fulfils its multifunctional roles in plants through the cascades of transcriptional responses of type-A RRs and type-B RRs [87,100]. Similar to *Arabidopsis*, a negative regulatory function of type-A RRs in CK-induced responses have also been observed in rice [101]. Notably, the unique expression of rice *OsRR6* was significantly induced by drought and ABA treatment. Furthermore, in the exogenous application of CKs, a strong positive correlation between transcription of *OsRR6* and yield was found in rice, opening the possibility that OsRR6 could participate in yield improvement under drought conditions [102]. The mechanism of how CK signaling functions in drought tolerance is often explained as directly cross-talking with ABA—a key hormone in abiotic stress response. Strong lines of evidence of the antagonistic action of CK-related RRs in ABA-response come from several findings involving sucrose nonfermenting 1-related kinases (SnRKs), which are key kinases in the ABA signaling pathway [62]. In this study, transcriptional analysis illustrated that SnRK2s act upstream of type-A ARR5 but downstream of type-B ARR1, ARR11 and ARR12, thus regulating ABA response and drought tolerance in *Arabidopsis* [62]. Thus, CK signaling genes can be highly selected and targeted in breeding programs on the enhanced plant drought tolerance.

### 2.3. CK Homeostasis and Signaling Components in Drought Tolerance

#### 2.3.1. CKs Function as Both Positive and Negative Regulators in Drought Stress Adaptation

The recent studies on CK homeostasis present CKs as having both positive and negative regulatory functions in plant adaptation to drought stress [103]. Many *in planta* studies have demonstrated the negative regulatory function of CK in drought stress response, as CK-deficient plants showed a higher ability of survival and tolerance under water deficit conditions [74,75,76]. For example, the overexpression of *AtCKX1* and *AtCKX3* resulted in the enhancement of root elongation and lateral root development, and leaf mineral enrichment, as well as drought tolerance in the transgenic *Arabidopsis* and tobacco (*Nicotiana tabacum*) [104]. In addition, advanced research in CK-deficient *Arabidopsis* plants with the *ipt 1 3 5 7* mutant genotype also showed drought-tolerant traits of these mutants [105]. Other established mutants of CK signaling components, such as *AHP2/3/5* [106], *AHK3* [107], and *ARR1/10/12* [87] mutants, consistently confer phenotypes of improved drought tolerance. Meanwhile, other research has reported that the overexpression of *ARR22* could improve the drought tolerance of transgenic *Arabidopsis*, by maintaining cell membrane integrity and inducing the expression of drought-responsive genes [108]. Interestingly, increasing CK endogenous levels through the expression of the *IPT* genes under the control of an appropriate promoter (e.g., *SAG*, *SARK*) could also enhance the drought tolerance in transgenic cotton (*Gossypium hirsutum*) [109], creeping bentgrass (*Agrostis stolonifera*) [110], eggplant (*Solanum melongena*) [111] and tropical maize [112]. Furthermore, studies on phytohormone secretions by drought-tolerant rhizobacterial strains and endosymbiotic *Methylobacterium oryzae* revealed the involvement of CKs in plant response under osmotic stress conditions [113,114]. According to these findings, application of these PGPB (plant growth promoting bacteria) would be beneficial in improving drought tolerance in plants. Therefore, although these are clear evidence to support the claim that targeting CK-related genes can improve plant adaptive response to drought, the obvious questions remain—how, and in what order, do CK metabolism and signaling complexes act together under drought? In the following section, we discuss the current understanding of the elaborated mechanisms that allow plants to survive drought stress through CK homeostasis in the context of ABA signaling responses.

#### 2.3.2. CK Actions in Drought Stress Response are Controlled by Hormone Crosstalk Regulation

**Figure 2:**
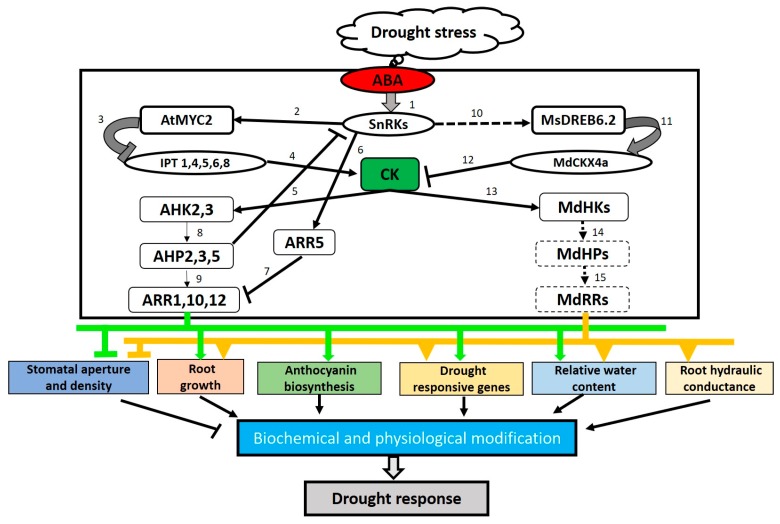
Proposed model of the regulatory functions of cytokinin (CK) signaling pathway in plant drought adaptation. Information for this pathway was suggested from previous studies [116,117]. Numbers represent regulation processes that were found in *Arabidopsis* (1-9) and apple (10-15). Dashed lines indicate proposed processes that have not been experimentally validated. Arrowheads represent activation, and perpendicular bars indicate inhibition. CK signaling in drought stress response induced by abscisic acid (ABA) can be divided into two unique mechanisms at the cellular level. One is the indirect regulation by ABA-responsive transcription factors [MYC (myeloblastosis) and DREB (dehydration-responsive element binding)] via the CK metabolic gene family members *IPT*s and *CKX*s, to initiate the reduction of endogenous CK levels in plant. In the other mechanism, the inhibition of CK action can be induced by ABA-responsive component SnRK2, which directly phosphorylates type-A RR5 (ARR5), a negative regulator of CK signaling. As a result, expression of stress-responsive genes is regulated to confer modifications in physiological and biochemical activities in mediating plant responses to drought conditions.

The role of ABA as a key signaling molecule in plant drought stress response is well-known, whereby ABA is accumulated under stress conditions and regulates the expression of ABA-responsive genes that are involved in a broad spectrum of biological functions [115]. Moreover, several ABA-related genes encoding MYB (myeloblastosis) or DREB (dehydration-responsive element binding) transcription factors (TFs) have been found to regulate intracellular pathways that impact CK homeostasis [116,117]. In *Arabidopsis*, an AtMYB2 knockout resulted in the up-regulation in *IPT1*, *4*, *5*, *6*, *8* expression, indicating the involvement of AtMYB2 in CK biosynthetic activities [116]. Findings from a study on wild apple showed that overexpression of *Mallus sieversii MsDREB6.2* led to the up-regulation of the *MdCKX4a* gene, thus reducing endogenous CK levels and improving the drought tolerance in transgenic plants [117]. Recently, a key player of ABA response, SnRK2 protein, was found to act upstream and directly interact with a negative RR of CK signaling—type-A ARR5, in mediating ABA response and drought tolerance in *Arabidopsis* [62]. Thus, it is clear that the involvement of CKs in plant drought adaptation is related to the antagonistic action of ABA, which can be explained by indirect interaction of ABA-responsive TFs with CK metabolic genes, and their direct interaction with CK signaling components. In this regard, the reduction in CK levels enables plants to cope with water deficit, through a wide range of morphological and biochemical changes [118]. These data suggest that the specific modulation of root/shoot ratio brings critical advantages to mutant plants (as summarized in Figure 2). Inhibition of CK signaling by ABA pathway may lead to the effective allocation of nutrient resources for root development, thus enhancing water access ability [104,117]. The reduction in CK content can also decrease stomatal aperture and density, increase root hydraulic conductance [117] or help maintain cell membrane integrity [105], hence contributing to plant tolerance to drought.

The known mechanisms and functions of the CK biosynthesis pathway in drought response suggest a remarkable strategy for developing drought tolerant crops, by inducing the reduction of CK content through root-specific CK gene expression, or using stress inducible promoters [74,75,76]. Furthermore, CK-related RRs were found to target multiple hormone signaling genes for auxin, ET, BRs and gibberellins (GAs) [90,119] in shoot development, providing more evidence for the interactions of CKs with other hormones during water stress response. Several independent studies characterized the potential association between CKs and JA under drought condition [120], but their mechanisms remain elusive. 

## 3. CK Modulation of Plant Physiological Characters to Mediate Drought Tolerance

### 3.1. CKs Modify Root Architecture and Improve Root Fitness

CKs modulate plant growth through changes in plant morphology and metabolism [104,121,122,123,124], acting as the indirect factors to limit damage caused by drought stress, hence increasing the chance of plant survival under water shortage conditions (Table 1) [39,44,125,126,127,128,129,130]. Abundant evidence exists to indicate that CKs play important roles in root growth and development in different plant species. Previous findings suggest that root growth and the development of rootstock are mainly influenced by sugar metabolism as well as auxin- and CK-signaling pathways [104,123]. In particular, the root-specific reduction of CK production in transgenic barley (*Hordeum vulgare*) carrying *bGLU::AtCKX1* [74] or *EPP::AtCKX1* [123], and in transgenic tobacco harboring *WRKY6::AtCKX1* [104] led to the enlarged root system in these transgenic plants. The increase of CK degradation activity was found to have positive effects on the number and length of lateral roots and root biomass accumulation, without penalties in shoot growth or seed yield [104,123]. Furthermore, in addition to the increased root size, reduction in the endogenous CK level in roots also resulted in higher concentrations of macro- and micro-elements, especially those with low soil mobility, such as phosphorous (P), manganese (Mn) or zinc (Zn) [123,130]. It has also been shown that the presence of elevated CK levels, either by ectopic expression of *IPT* or by the exogenous treatment of CKs, could result in the increased transcriptional level of *CKX* genes and/or CKX activity. Positive correlations between the expression patterns of *IPT* and *CKX* GFMs have been reported during seed develpment of *B. napus* [131], early maturation stage of *B. rapa* [132], carpel and seed development [133], and kernel development in wheat (unpublished data) and in maize kernels [35].

On the other hand, an enhanced root system with less cellular damage in transgenic plants compared with the wild-type plants under drought conditions could also be achieved through increasing CK levels by using an inducible promoter to drive the expression of *IPT* target gene [109,110]. Another defense mechanism that has been employed by this approach is the delay of drought-induced senescence [109,111,112]. Taken together, we suggest that the mechanisms enhancing drought tolerance by the up- or down-regulation of endogenous CK levels might involve different pathways and crosstalk with other phytohormones. For example, CK signaling was found to regulate auxin-efflux and influx carriers [134,135], which are important in controlling root developmental processes, such as root formation, emergence, elongation and gravitropism [136]. Elucidating these pathways could provide significant insights into the roles of CKs in drought stress, which could ultimately contribute to the development of CK-mediated, drought-tolerant crops.

### 3.2. CKs Influence Photosynthetic Machinery

Photosynthesis is negatively affected by drought stress, primarily through stomatal closure and metabolic impairment [137]. CKs, with their impact on different levels of photosynthetic machinery [138], can compensate for the decrease in photosynthetic rate caused by water-deficit conditions, through modulating stomatal conductance [139] or chlorophyll biosynthesis [140]. Previous findings also support the assumption that increased endogenous CK levels can enhance plant photosynthetic rates under drought. Transgenic cotton and peanut carrying *P_SARK_::IPT* had higher chlorophyll content, photosynthetic rates and/or higher stomatal conductance and transpiration under water-deficit conditions, compared with the WT plants [38,109]. Higher chlorophyll content was also observed in *P_SAG12_::IPT*-transgenic creeping bentgrass experiencing drought [126]. 

The prevention of photosynthetic machinery degradation might be a result of the activation of BR-associated pathways, which are positively regulated by increased CK levels in *IPT*-transgenic tobacco plants [141]. Recent studies indicated that BRs promote CO_2_ assimilation and quantum yield of photosystem II (PSII) [142], as well as the protection of PSII in plants treated with herbicides [143]. Furthermore, CKs can also stimulate the production of photosynthetically active pigments [140] involved in the light-dependent phase of photosynthesis, as well as the key enzymes of the light-independent phase [138]. Moreover, other CK-mediated mechanisms involved in maintaining photosynthesis under drought stress might also exist, thus further investigations are necessary.

### 3.3. CKs Modulate Plant Water Balance

Effective water management under water-deficit stress is crucial for overcoming water shortage conditions. Along with the increasing root size that allows easier access to a water source, limiting the rate of water loss through stomatal activities also plays an important role in plant adaptation to drought. CKs have been found to play regulatory roles in different processes that relate to stomata, such as stomatal conductance [139] and stomatal density [144].

Low CK levels generally have a positive effect on plant water status, as observed in transgenic plants overexpressing CK dehydrogenase genes [75,144]. This could be the result of an enlarged root system [74,119], lower stomatal conductance [75] or stomatal density [144]. Research on apple (*Malus × domestica*) revealed another approach to acquire similar benefits for the transgenic plants, as evidenced by the overexpression of the dehydration-responsive gene *MsDREB6.2*, leading to the induction of *MdCKX4a* expression [117].

Again, increasing endogenous CK levels through the controlled expression of *IPT* GFMs can result in similar effects, as demonstrated in transgenic plants of different species with better capacity of maintaining cellular relative water content, such as creeping bentgrass using senescence-activated promoter *P_SAG12_* [126], and tropical maize [112] and sweet potato (*Ipomoea batatas*) [145], using water-deficit responsive and maturation-specific promoter *P_SARK_*. This acquisition is suggested to be caused by the CK delaying the normal drought-induced leaf senescence in the transgenic plants [145].

Furthermore, the crosstalk between CKs and other phytohormones, especially ABA, which regulates stomatal activity, and ET which regulates leaf senescence, is important for clarifying the mechanism of CK-mediated maintenance of water status in plants under drought stress. It has been found that RRs in the CK signaling pathway can be phosphorylated by SnRK2s, a key regulator of stomatal activity that belongs to the ABA signaling pathway [62]. Similarly, the CK-related RRs were also found to interact with components of ET signaling pathway, such as ERF1 (Ethylene-responsive factor 1) and EIN3 (Ethylene-insensitive 3) [146]. This could be a breakpoint in the further understanding of the correlations between these phytohormones in drought stress, that will help in achieving drought resistance in plants.

### 3.4. CKs Enhance Antioxidant Defense Systems

Reactive oxygen species (ROS), by-products of the photosynthesis process, which are rapidly accumulated under stress conditions, can cause major oxidative damage to the cell membrane, proteins, DNA and RNA molecules [147]. CKs affect the accumulation of ROS through different mechanisms, such as inhibiting activities of ROS-generated enzymes (e.g., xanthine oxidase) or increasing activities of antioxidant enzymes (e.g., superoxide dismutase (SOD) and catalase (CAT)) [138,148].

A number of studies have demonstrated multiple molecular correlations between CKs and antioxidant enzyme activities through exogenous CK treatment or through experimentally enhanced endogenous CK levels via transgenic approaches. In this context, the excess of CKs in plant often affects cellular processes, that are not otherwise under the regulation of CKs. Interestingly, using a reverse approach, it has been shown that reduced CK levels affect activities of antioxidant enzymes during the life span of tobacco [149]. For example, transgenic tobacco overexpressing *Arabidopsis AtCKX2* under the control of a constitutive *35S* promoter revealed enhanced activities of SOD, glutathione reductase (GR) and ascorbate peroxidase (APX) [149]. Consistently, the overexpression of *AtCKX1* under the control of root-specific promoter *WRKY6* or constitutive promoter *35S* also promoted the expression of genes that encode the antioxidant enzymes, such as CAT, APX or SOD in transgenic tobacco [76]. Moreover, the ectopic expression of *CKX* in barley via a mild root-specific promoter (maize *β-glucosidase*) has also been found to alter root architecture and led to stronger lignification of the root tissue as well as activate the biosynthesis of flavonoids [75], non-enzymatic antioxidant participants in plant drought stress tolerance mechanisms [150].

Creeping bentgrasss, overexpressing an *Agrobacterium IPT* gene under the control of the senescence-activated promoter *SAG12*, exposed to drought stress exhibited significantly lower ROS content, accompanied by higher antioxidant enzymatic activities of SOD, CAT, ascorbate peroxidase (APX) and dehydroascorbate reductase (DHAR), compared with those in the non-transgenic plants [126]. A similar construct transformed into eggplant also revealed the enhanced activities of ROS-scavenging enzymes in the transgenic plants [111]. The positive effects of CKs on antioxidant defense systems might be related to the delayed drought-induced senescence and enhanced photosynthetic characteristics, which have been elaborated in Section 3.2. 

It is therefore apparent that CK-mediated plant tolerance to drought could be achieved through two seemingly contradictory approaches. This is probably due to the choice of promoter and type of tissue modulated for transgene expression, which led to the regulation of different pathways. However, the detailed mechanisms remain elusive and require further investigations for more insights into CK involvement in plant defense systems.

### 3.5. CKs Affect Drought-Responsive Gene Expression

Drought-responsive genes play important roles in plant adaptation under drought stress conditions. However, the molecular mechanisms related to CKs behind this process remain uncertain [151]. Previous genome-wide analyses have shown that altering the endogenous CK levels by modulating the expression of either *IPT* or *CKX* genes could affect the expression of several sets of genes involved in different processes that could play major roles in improved drought tolerance. Examples of these various processes include energy production, metabolic activities, stress defense, signaling, protein synthesis and transport, and membrane transport [74,75,126,151]. Molecular analyses in creeping bentgrass overexpressing CK biosynthesis-related gene *IPT* from *A*. *tumefaciens* under the control of the *SAG12* promoter revealed the expression of genes encoding proteins with different functions such as CAT (ROS detoxification), RuBisCo large subunit (energy production), Leu-rich repeat (LRR) receptor-like kinase (transmembrane receptor proteins), oxygen-evolving enhancer protein 3–1 and chloroplast precursor (OEE3) (metabolism), and universal stress protein 5327 (stress defense) [151]. In addition, transgenic creeping bentgrass harboring *P_SAG12_::IPT* showed induced the expression of TF-encoding genes such as *bHLH148*, *MYB4/4*-like, and *WRKY28/53/71* under drought, which can lead to the initiation of defensive responses, including protein modification and degradation, RNA degradation, or JA- and ABA-regulated responses [126]. In another study, roots of transgenic barley overexpressing *AtCKX1* gene under drought stress revealed a higher abundance of transcripts involved in phenylpropanoid pathway, phenylalanine synthesis, photosynthesis and drought-responsive TFs [75]. As mentioned above, these CK-responsive genes might interact with/belong to pathways under the control of other phytohormones such as ABA, JA, auxin, BRs and ET. Altogether, these ultimately form a global defensive system mediating plant responses to drought. It is crucial to complete these transcriptional dynamic networks to fully understand the plant strategy of adaptation to adverse environmental conditions and provide essential knowledge for obtaining drought-resistant crops in the future.

## 4. Conclusions and Future Directions

We anticipate that the CK-related mechanisms will be widely investigated in modern plant breeding programs for drought tolerance, both from a fundamental as well as from an application perspective. Indeed, transgenic CK modifications are already being actively implemented in crop plants, because of their strong effects on seed yield improvement. Understanding of the thus-far overlooked functions of CK metabolic and signaling genes in drought stress response, such as in crosstalk with other signaling pathways and the identification of genes under CK regulation, will improve the basic knowledge of plant hormonal biology, allowing the selection and *in planta* characterization of potential new regulators for plant drought adaptation. Although recent studies have shed new light on several genetic signaling components required for CK functioning and their crosstalk with ABA, many questions regarding CK-induced alterations of various physiological processes relating to vascular cell differentiation for root fitness improvement or the inter-organ communication network remain elusive. Further studies on spatiotemporal specific gene expression and cell developmental trajectories are required to address these questions.

Overall, CKs play a pivotal role in plant response to drought stress. The expected unravelling of the CK signaling networks and their crosstalk with numerous biochemical pathways will draw a detailed picture of fascinating progress—a road towards developing drought-tolerant crops, and in the long-term, a more sustainable agriculture.

## Figures and Tables

**Table 1:** Summary of cytokinin (CK) modification-related studies and their reports on corresponding drought-tolerant phenotypes.

CK Metabolic Gene and Source of Isolation	Genetic Engineering Approach	Promoter Controlling Transgene Expression	Transgenic Species	Phenotype Alterations	References
*AtCKX1* (*Arabidopsis thaliana*)	overexpression	*Beta-glucosidase* (*bGLU*) from maize	Barley (*Hordeum vulgare*)	maintain higher water content; enhance growth and yield; increase root growth; alter drought-responsive gene expression; improve drought stress tolerance	[74,75]
	overexpression	root-specific promoter *WRKY6* and constitutive promoter *35S*	Tobacco (*Nicotiana tabacum*)	maintain higher expression levels of genes encoding antioxidant enzymes and improve drought stress tolerance	[76]
*IPT* (*Agrobacterium tumefaciens*)	inducible expression	stress- or senescence-activated promoter *SAG12*	Creeping bentgrass (*Agrostis stolonifera*)	alter transcriptional factor-encoding genes involved in stress signaling, oxidative protection and protein modification; enhance drought tolerance	[125,126]
*IPT* (*Agrobacterium tumefaciens*)	inducible expression	stress- and maturation-induced promoter (*SARK*)	Rice (*Oryza sativa*)	enhance sink strength; improve drought tolerance and increase grain yield	[56]
*IPT* (*Agrobacterium tumefaciens*)	inducible expression	stress- and maturation-induced promoter (*SARK*)	Peanut (*Arachis hypogaea*)	maintain higher photosynthetic rates, stomatal conductance and transpiration; improve drought tolerance and increase yield under field conditions.	[38]
*IPT* (*Agrobacterium tumefaciens*)	overexpression	modified developmentally regulated transcription factor *AtMYB32* (AT4G34990) promoter from *Arabidopsis*	Canola (*Brassica napus*)	increase higher chlorophyll levels; delay leaf senescence; enhance yield under rain-fed and irrigated conditions	[127]
*IPT* (*Agrobacterium tumefaciens*)	inducible expression	stress- and maturation-induced promoter (*SARK*)	Cotton (*Gossypium hirsutum*)	delay senescence; enhance root and shoot biomass; maintain higher chlorophyll content and photosynthetic rates under water deficit conditions	[109]
*IPT* (*Agrobacterium tumefaciens*)	inducible expression	stress- and maturation-induced promoter (*SARK*)	Rice (*Oryza sativa*)	increase drought tolerance through the coordinated regulation of carbon and nitrogen assimilation	[39]
*IPT* (*Agrobacterium tumefaciens*)	overexpression	modified developmentally regulated transcription factor *AtMYB32* (AT4G34990) promoter from *Arabidopsis*	Wheat (*Triticum aestivum*)	increase grain yield under water deficit	[44]
